# Ischemic Stroke Secondary to Meningovascular Syphilis

**DOI:** 10.1177/23247096261454453

**Published:** 2026-05-19

**Authors:** Marissa Nicolas-Cagigal, Aaron Lee, Juan C. Sarria

**Affiliations:** 1 John Sealy School of Medicine, 74950 University of Texas Medical Branch, Galveston, TX, USA; 2 Division of Infectious Diseases, Department of Internal Medicine, 223232 University of Texas Medical Branch, Galveston, TX, USA

**Keywords:** syphilis, meningovascular, ischemic stroke, HIV

## Abstract

Syphilis can present in a wide variety of ways, earning its reputation as “the great imitator”. In a minority of cases, the bacteria can invade the central nervous system and produce meningovascular syphilis, an inflammatory vasculopathy that can mimic neurovascular disease by manifesting as an ischemic stroke. Consideration and timely recognition of this manifestation of syphilis infection is paramount because if left untreated treponemal induced arteritis can progress, keeping patients at increased risk for additional strokes. We report the case of a 36-year-old man whose untreated HIV placed him at increased risk for the development of meningovascular syphilis. We also describe the use of an acceptable alternative therapy after the patient developed an allergic reaction to penicillin during treatment. This case highlights the importance of maintaining a high level of suspicion for infectious etiologies in patients presenting with stroke-like symptoms without conventional risk factors for neurovascular disease. In this case, such suspicion enabled a timely diagnosis and treatment, preventing further complications of untreated syphilis.

## Introduction

Meningovascular syphilis is an early form of neurosyphilis characterized by meningeal inflammation and endarteritis caused by untreated systemic *Treponema pallidum* infection.^1^ On average, patients with this form of neurosyphilis have lived with the infection for about seven years before developing meningovascular sequelae. This form of infection accounts for only 11% of neurosyphilis cases worldwide.^2^

Meningovascular syphilis is a result of inflammation of small- and medium-sized arteries of the brain, which eventually leads to fibroblast activation. This perivascular inflammation and fibrosis promote intimal thickening of the affected vessels, narrowing the lumen.^2^ Regardless of age or other comorbidities these patients are at increased risk for thrombotic cerebrovascular events due to this process.^3^ Prodromal symptoms typically include headaches, personality changes, or cranial neuropathies, and are eventually followed by an ischemic stroke, which is the most common manifestation of this form of infection.^4^

Diagnosis relies on a thorough history and high clinical suspicion in high-risk individuals. Although not always present, if CSF analysis reveals positive Venereal Disease Research Laboratory (VDRL) serology this can confirm the diagnosis.^3^ As CSF VDRL is the most specific diagnostic tool for meningovascular syphilis. This manifestation of syphilis commonly presents in patients with comorbid HIV, which can accelerate progression and alter clinical presentation. HIV co-infection not only increases susceptibility to neurosyphilis but may also reduce the reliability of traditional serologic and CSF markers, further complicating diagnosis and treatment decisions.^2^ Here we present a CSF VDRL-confirmed case of meningovascular syphilis in a patient with newly diagnosed HIV and discuss the successful use of an alternative antibiotic regimen for treatment.

## Case Presentation

A 36-year-old male with no known past medical history was brought to the emergency department due to altered mentation that had started 16 hours prior to presentation. On arrival, the patient was noted to have left lower extremity weakness. He was unable to provide any history as he was confused, inattentive, and exhibiting mutism, only occasionally answering questions, mostly with strange responses. On arrival he had a temperature of 36.7°C and blood pressure of 147/93 mm Hg. On physical examination, the patient was oriented only to self. He was observed fidgeting and appeared agitated and withdrawn. He was noted to have left lower extremity weakness, with his strength graded as 3/5; all other extremities were graded as 5/5 strength.

Laboratory studies were significant for sodium 131 mmol/L, calcium 8.2 mg/dL, blood glucose 114 mg/dL, ammonia < 9 µmol/L, AST 45 U/L, and HIV testing was reactive. Later he was found to have a viral load of 318,382 copies/ml and an absolute CD4 count of 71 cells/µL.

**Figure 1.**
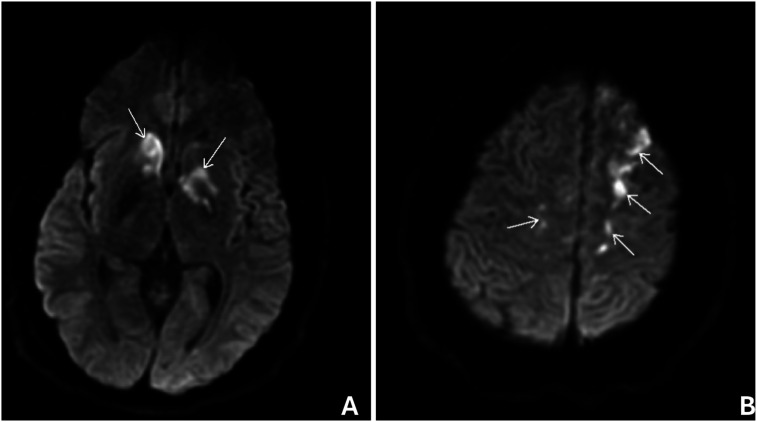
Axial diffusion-weighted magnetic resonance imaging of the brain. The arrows indicate areas of hyperintensity, representing restricted diffusion. These findings are consistent with acute to subacute ischemic infarcts involving the bilateral basal ganglia (A) and frontal lobes, with greater involvement of the left hemisphere (B)

Due to acute onset of altered mental status and left lower extremity weakness, stroke activation was initiated. A computed tomography (CT) scan of the head showed no acute intracranial hemorrhage or mass effect. CT angiogram of the head and neck showed no high-grade stenosis or aneurysm in the intracranial or cervical vessels. Magnetic resonance imaging demonstrated infarcts of the bilateral basal ganglia and frontal lobes with distribution most suggestive of watershed infarcts (Figure 1A and B).

The patient was started on amlodipine 5 mg daily, aspirin 81 mg daily, and atorvastatin 40 mg nightly. Due to ischemic stroke in the setting of low risk profile and new diagnosis of HIV a syphilis workup and lumbar puncture were recommended. Syphilis IgG/IgM was reactive and his RPR was 1:512. The patient was then started on penicillin G 4 million units every 4 hours and bictegravir-emtricitabine-tenofovir alafenamide daily. A lumbar puncture was performed with an opening pressure of 25 cm H_2_O. CSF analysis was significant for WBC 47 cells/μL with a lymphocyte predominance, RBC 281 cells/μL, glucose of 32 mg/dL, and total protein of 398 mg/dL. About 9 days later, the CSF VDRL was reported as reactive.

On day 7 of penicillin therapy, the patient developed diffuse non-erythematous, non-pruritic nodules over the back, abdomen, and bilateral upper extremities. On day 10 of treatment, the rash became more pronounced, erythematous, and pruritic. He was switched to ceftriaxone 2 g IV once a day. Ultimately, the patient’s treatment course consisted of 9 days of penicillin G followed by 6 days of ceftriaxone. Upon discharge, the decision was made to complete treatment with 14 days of doxycycline 100 mg BID instead of the standard penicillin G benzathine intramuscular injection. Throughout the admission his mentation waxed and waned, by the time of discharge, he was alert and oriented to self, location, and time. On an 8-month telemedicine visit, the patient reported feeling well and denied any neurologic symptoms. Unfortunately, the patient did not follow up in clinic for further evaluation or testing.

## Discussion

### Epidemiology

Neurosyphilis, including meningovascular syphilis, is most prevalent among populations that are at high risk of contracting HIV. Of particularly high risk, are patients with untreated HIV, low CD4 counts, or those who have a detectable viral load.^5,6^ The explanation for the association between these two infectious diseases is likely multifactorial. First, they share the same high risk sexual behaviors. Additionally, patients who have poorly treated HIV may have barriers to care that might also allow syphilis to go undiagnosed and untreated, increasing their risk of developing neuroinvasive disease**.** Finally, poorly managed HIV weakens an individual’s immune system which could make neuroinvasion easier.^5,7,8^

### Typical Presentation

The most common presentation of meningovascular syphilis is a young adult with an acute ischemic stroke followed by significant post-stroke disability.^9,10^ Previous studies have found that patients with syphilis-related strokes experienced significantly greater stroke-related disability than patients with strokes unrelated to syphilis.^2,8,10^ Additionally, these patients may present with an acute ischemic stroke in the absence of atherosclerotic risk factors.^11^ Ischemic strokes secondary to meningovascular syphilis are most commonly located within the middle cerebral or vertebral-basilar artery territories.^9,12^ In addition to evidence of infarcts, MRI may also show meningeal enhancements and arterial irregularities. Angiographic studies can demonstrate arteriopathy, showing narrowing or even total occlusions of affected vessels. Often syphilis-related strokes are preceded by other neurologic symptoms such as headaches, dizziness, mood swings, decreased visual acuity, memory loss, or altered mentation.^9^

### Diagnostics

CSF analysis provides a foundation for a definitive diagnosis of neurosyphilis.^3^ A CSF profile suggestive of neurosyphilis involves an increased WBC count, typically 20 cells/μL or more, and an increased CSF protein, typically greater than 50 mg/dL.^13,14^ Further analysis of CSF can include treponemal and nontreponemal tests. Treponemal tests have high sensitivity but are not specific for neurosyphilis due to passive diffusion along the blood brain barrier (BBB).^3,15^ Thus, the utility of treponemal tests are most valued for their ability to rule out a diagnosis of neurosyphilis when negative.^16,17^ Instead, the CSF VDRL assay detects large molecules that do not cross an intact BBB, making it highly specific and therefore the single most reliable test for neurosyphilis. In summary, a negative CSF VDRL cannot rule out neurosyphilis, but a positive CSF VDRL can confirm the diagnosis.^3,18,19^

### Management of Meningovascular Syphilis

All patients diagnosed with neurosyphilis should be offered HIV testing at the time of diagnosis. Patients who test negative should be offered HIV pre-exposure prophylaxis, and those who test positive should be initiated on antiretroviral therapy (ART) without delay. Regarding the treatment of neurosyphilis, the recommended regimen includes aqueous crystalline penicillin G 18-24 million units per day for 10-14 days, which can be administered as 3-4 million units IV every 4 hours or as a continuous infusion. This can be followed by benzathine penicillin 2.4 million units IM weekly for 1-3 weeks. This regimen is recommended to all patients regardless of HIV status.^20^

Follow up testing to monitor treatment response should include a serum RPR. The frequency of this serologic evaluation is determined by the stage of infection. Meningovascular syphilis is an early form of neurosyphilis; for these patients, serologic testing is recommended at 6 and 12 months after treatment.^8^ Follow up RPR should be compared to the titer at the time of treatment. Adequate treatment of neurosyphilis is represented by normalization of the RPR, defined as either a four-fold decrease in RPR or a non-reactive RPR. Failure of the RPR to normalize suggests treatment failure and requires retreatment. Normalization of the RPR can predict normalization of CSF parameters, thus it is not necessary for patients without HIV or patients with HIV on ART to repeat CSF analysis. Instead, treatment response should be monitored clinically and serologically.^20^

It is important to note that some data suggest delayed normalization of CSF parameters in patients with HIV, especially if there is advanced immunosuppression. Additionally, within this group of patients there is concern for greater risk of treatment failure. For these reasons, these patients should be monitored more frequently. Recommendations for patients with HIV and early neurosyphilis include RPR monitoring at 3, 6, 9, 12, and 24 months after treatment. In patients with HIV, the RPR is expected to demonstrate a four-fold decrease within 12-24 months, failure to do so is indicative of treatment failure and requires retreatment. Additionally, retreatment is indicated for all patients regardless of HIV status if at any time there is persistence or recurrence of neurologic symptoms or a four-fold increase in RPR for two weeks or longer. Retreatment should include the same IV penicillin based neurosyphilis regimen used for initial treatment, as previously discussed.^20,21^

### Encountering Penicillin Hypersensitivity

Penicillin G remains the first-line treatment for all forms of neurosyphilis, including meningovascular syphilis.^22^ However, pharmacological management of neurosyphilis can be complicated by penicillin hypersensitivity. Ceftriaxone has been shown to provide clinical efficacy as an alternative therapy in the treatment of neurosyphilis in many of these patients.^23,24^ The typical recommendation for this alternative regimen includes ceftriaxone 2 g IV once a day for 10-14 days.^3^ Additionally, classic neurosyphilis regimens conclude with a benzathine penicillin G injection, however an accepted alternative is doxycycline 100 mg twice daily for 4 weeks in patients with serious penicillin hypersensitivity.^13,23-25^

## Conclusion

This case emphasizes the importance of maintaining a high index of suspicion for neurosyphilis in young patients presenting with unexplained strokes. While penicillin remains the gold standard for neurosyphilis treatment, ceftriaxone can serve as a safe and effective alternative in patients who develop hypersensitivity reactions. Timely recognition and appropriate antimicrobial therapy are critical for optimizing outcomes in this potentially debilitating but treatable condition.
